# Does bird life-history influence the prevalence of ticks? A citizen science study in North East Spain

**DOI:** 10.1016/j.onehlt.2024.100718

**Published:** 2024-04-03

**Authors:** Jesus Veiga, Oriol Baltà, Jordi Figuerola

**Affiliations:** a Facultad de Farmacia, Universidad de Granada, Granada, Spain; b Institut Català d'Ornitologia, Barcelona, Spain; c Estación Biológica de Doñana – CSIC, Sevilla, Spain; d CIBER Epidemiology and Public Health (CIBERESP), Madrid, Spain

**Keywords:** Tick prevalence, Host-pathogen interactions, Parasite incidence, Migrant birds, Long distance dispersal, Gregariousness, Long-term study

## Abstract

After mosquitoes, ticks are among the most important vector of pathogens of concern for animal and public health, but unless mosquitoes ticks remain attached to their hosts for long time periods providing an opportunity to analyse their role in the dispersal and dynamics of different zoonotic pathogens. Given their interest in public health it is important to understand which factors affect their incidence in different hosts and to stablish effective surveillance programs to determine the risk of transmission and spill-over of zoonotic pathogens. Taking benefit of a large network of volunteer ornithologists, we analysed the life-history traits associated to the presence of ticks using information of 620,609 individuals of 231 avian species. Bird phylogeny, locality and year explained a large amount of variance in tick prevalence. Non-colonial species non breeding in grasslands and non-spending the non-breeding season as gregarious groups or isolated individuals (e.g. thrushes, quails and finches) had the higher prevalence of ticks and appear as good candidates for zoonosis surveillance programs based on the analyses of ticks collected from wild birds. Ringers underestimated tick prevalence but can be considered as an important source of information of ticks for public and animal health surveillance programs if properly trained for the detection and collection of the different tick development phases.

## 1 Introduction

Ticks are vectors of numerous pathogens including, bacteria, viruses and protozoa, that may affect human and wildlife health (see [1]). Birds are known reservoirs of several tick-borne pathogens with medical and veterinary importance [2,3] such as Crimea-Congo haemorragic fever virus, bacteria of the *Borrelia burgdorferi* sensu lato (s.l.) complex and protozoans of the genus *Babesia* [4]. Understanding the transmission of such pathogens requires an understanding of the ecological and environmental factors that affect pathogen amplification, vector abundance and distribution and their interactions with the vertebrate hosts [5,6]. Several species of ticks are expanding their distribution in the Palearctic [7] and birds have been proposed as an important tick carrier and their pathogens to new locations [8]. Several studies done in southern [9] and northern [10] Europe have shown the presence of ticks on migratory birds, supporting their role in long distance tick dispersal. In fact, Norte et al. [11] concluded that the population structure of *B. burgdorferi* s.l., is determined by the association of ticks with bird hosts and their movements. Therefore, birds are crucial for tick dispersal and pathogen transmission cycles, acting as reservoirs for a plethora of infectious agents [12,13]. Thus, identify bird species more exposed to ticks help to understand pathogen amplification cycles but also to set up efficient surveillance programs to detect pathogen dispersal and intensity of circulation. More than 540 bird species occurred regularly and naturally in Europe [14] and consequently applying a functional traits approach [15], to identify the ecological and life-history traits (if any) that makes an avian species more exposed to ticks may contribute to improve surveillance efficacy.

Ticks prevalence varies spatially and temporally in relation with the interaction between avian host traits and tick phenology that enable host exposure [16]. For example, in the Brazilian Pantanal, the probability of an individual bird of being infested with ticks was higher for resident birds that forage at ground level and during the transition between the dry and wet season [17]. The variability in tick presence also depends on environmental conditions, with temperature and humidity affecting tick activity and distribution [18]. Therefore, tick prevalence could vary among years, seasons, localities and bird species [10]. The heterogeneity of tick prevalence together with their low prevalence makes difficult to determine the relevant factors affecting their distribution. Therefore, it is necessary to examine high numbers of birds over a long period of time. Nowadays, citizen science, which consist on involve citizens in scientific endeavour that generates knowledge [19], have proved to be truly useful to obtain high amount of observations and to produce valuable long-term information for scientific analysis [20]. Bird ringing is one of the longest running citizen science experience in the world, run by highly specialized participants with large knowledge of bird handling and identification but, usually, no experience in parasitology and entomology. In this study we take advantage of one of these programs in which ringers were requested to report the presence of ticks in the ringed birds, to explore how bird life-history traits predict the probability of being infested by ticks.

The aim of the study is to 1) identify the avian species with higher prevalence of ticks and 2) determine the life-history traits associated to tick presence. We predict that i) longer bills will promote higher prevalence of ticks due to lower preening efficiency; ii) longer distance migrants will had higher prevalence of ticks due to the exposition to a higher number of habitats; iii) traits associated with gregariousness will increase tick prevalence due to increased bird to bird transmission; iv) some habitats used by the different species will increase or reduce tick prevalence, due to major influence of habitat over tick abundance. Long-terms analysis together with a high sampling effort of different species is necessary to detect this kind of general patterns. Therefore, we take advantage of a protocol established in 2003 for detailed data collection during bird ringing that included the recording of tick presence in captured birds. We will also compare the tick prevalence found in this study by amateur ringers with other previous studies performed by trained entomologist analysing the incidence of ticks in birds, to detect the possible bias in tick detection using citizen science.

## 2 Methods

### 2.1 Data collection and curation

This study took place in Catalonia (Northeast Spain) where “Institut Catalá d'Ornitologia (ICO)” coordinates the activity of all amateur bird ringers in the region. The organization stablished different standard ringing procedures involving recording a different number of variables. One of this procedures included examining the birds to reveal the presence of ticks. Since 2003, 205 different bird ringers have provided information to the program recording the presence of ticks together with bird ring, age, sex (when possible), locality of ringing and location coordinates. The main bird sampling method consist of mist nest but other trap methods established in the “ICO Ringing Standards” as Yunick tarp, claptrap, Helgoland, feeder trap, and capture at the nest were also used. As most birds were captured by mist nest, we considered that most of recorded individuals were hard-ticks rather than soft-ticks. Soft-ticks typically feed in shelters for short periods time, mostly at night, and retreat after feeding to refugia in the vicinity of the host, which make unlikely the detection of soft ticks on bird capture.

The database includes 620,609 captures from 231 species sampled at 154 different locations from 2003 to 2020. Most captures were done within Catalonia and surroundings, but some far locations were also included in the database (i.e. Morocco). In order to avoid confounding factors, we selected the observations located in the UTM zone 31 T, and localities situated too close (less than 1 km of distance based on the coordinates) were merged into a single locality. We randomly selected one observation per individuals captured in more than one occasion (as determined from the ring number) with the function *slice_sample* from the *dplyr* R package [21]. Finally, we removed species with less than 15 individuals captured to increase the reliability of prevalence estimates [22]. The species *Cuculus canorous* was also removed because information was not available for all the life-history traits included in the analysis. The final database included 473,326 individuals from 127 species and 117 localities.

### 2.2 Life-history traits selection

Several life-history traits may affect tick prevalence. We selected 16 life-history traits variables of the 85 presented in the database produced by Storchová & Hořák [23]. In particular: i) mean bill size, ii) migration behaviour (sedentary, short-distance migrant or long-distance migrant), iii) association outside of the breeding season (gregarious, in pairs or solitary), iv) association during nesting (solitary, semi-colonial, colonial), v) territoriality (yes or no), vi) main habitat occupied in the breeding area (forest, shrub, grassland, mountain meadows, aquatic, rocks, human settlements). Some habitat levels were merged based on their similarities, specifically, forests include deciduous forests, coniferous forests and woodland and aquatic habitats includes reeds, swamps and freshwater (includes static and flowing freshwaters). As several species contain more than one level of each variable, these variables were transformed to dummy, namely migratory behaviour, association outside of the breeding season and during nesting, and habitats occupied.

### 2.3 Statistical analysis

To explore what life-history traits were associated to tick presence we fit a multivariate generalised linear mixed models using Markov chain Monte Carlo (MCMC) techniques following [24]. Uninformative priors equivalent to an inverse-gamma prior with shape and scale equal to 0.001 were used to avoid influence of priors on the posterior distribution estimation [25]. To control for phylogenetic relationships between avian species we used a working phylogeny. We download 1000 trees from BirdTree database [26], from the Hackett tree distribution. A consensus tree was build adopting a 50% majority-rule using SumTree v4.1.0 in DendroPy v4.1.0 [27,28] following Rubolini et al. [29]. The MCMC was run in four parallel threads of 2,500,000 iterations, with a burn-in of 40,000 and a thinning interval of 5000, resulting in 9,840,000 iterations and between 966 and 1887 samples of the posterior distribution parameters. As the analysis was run in four parallel threads, output values were calculated as the mean of the four estimates. In the fitted models, the presence of tick of each individual was the response variable and all the mentioned life-history traits were included as fixed factors (see above). Tick presence was modelled as a categorical variable and location, hydrological year (from September to August of the following year) and bird phylogeny were included as random factors. The identities of ringers were not included as random factor because this information was not provided. However, their influence is partly included within the location variable, as ringers often sample the same locations. We also explore spatial auto-correlation analysis by means of Moran coefficient using Delaunay triangulation to test if spatial autocorrelation must be considered in the analysis, but we did not obtain a significant effect (Moran-I = -0.033; *p*-value = 0.7). Phylogenetic signal λ was modelled as an equivalent to Pagel's λ model of phylogenetic signal inference [30]. An additional model without including the phylogeny but with the same permutation parameters (see above) was performed to detect if some life-history traits, even when dependent of the phylogeny, correlates with tick occurrence. Between 748 and 1869 samples of the posterior distribution parameters were obtained for this non phylogenetically controlled model.

## 3 Results

Ticks were detected in 646 out of 473,326 individuals (overall prevalence of 0.14%, CI95%: 0.13–0.15) belonging to 51.18% of the 127 avian species analysed. The ten species with highest prevalence were *Turdus iliacus* (3.06%, *n* = 98)*, Oenanthe oenanthe* (2.44%, *n* = 41)*, Coturnix coturnix* (1.64%, *n* = 61)*, Delichon urbicum* (1.32%, *n* = 302)*, Hippolais icterina* (1.10%, *n* = 91)*, Phoenicurus phoenicurus* (1.01%, *n* = 2369)*, Lullula arborea* (1.00%, *n* = 100)*, Emberiza cia* (0.95%, *n* = 423)*, Coccothraustes coccothraustes* (0.90%, *n* = 445) and *Turdus merula* (0.82%, *n* = 15,573) (Supplementary Table 1). From the 14 orders sampled, 7 were parasitized by ticks namely, Galliformes (1.18% prevalence, *n* = 85), Caprimulgiformes (0.30% prevalence, *n* = 33), Columbiformes (0.21% prevalence, 484 individuals), Strigiformes (0.19% prevalence, 540 individuals), Passeriformes (0.14% prevalence, 463,794 individuals), Piciformes (0.07% prevalence, 1355 individuals) and Coraciiformes (0.02% prevalence, 5385 individuals). No ticks were detected in species of the orders Accipitriformes (*n* = 108), Anseriformes (*n* = 103), Charadriiformes (*n* = 245), Falconiformes (*n* = 69), Gruiformes (*n* = 422) and Pelecaniformes (*n* = 406).

**Fig. 1:**
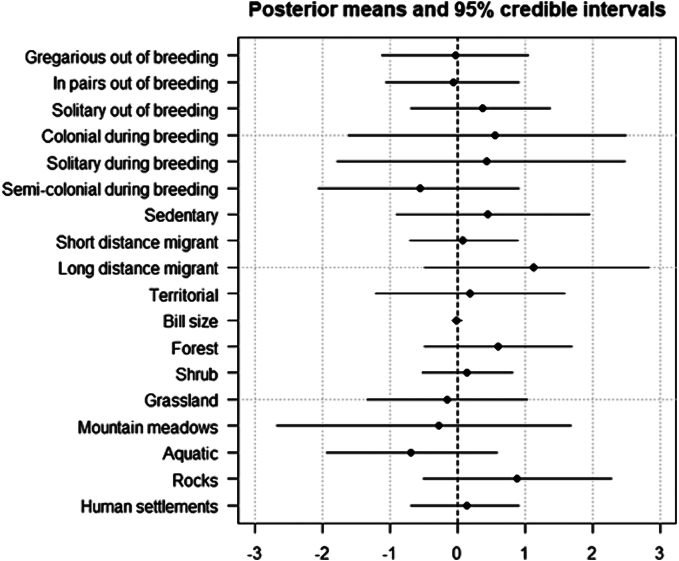
Posterior mean estimates and 95% credible intervals of fixed effects predictors of the probability of being infested by ticks when phylogeny is included in the model as a random factor. Intercept were removed for visualisation.

**Fig. 2:**
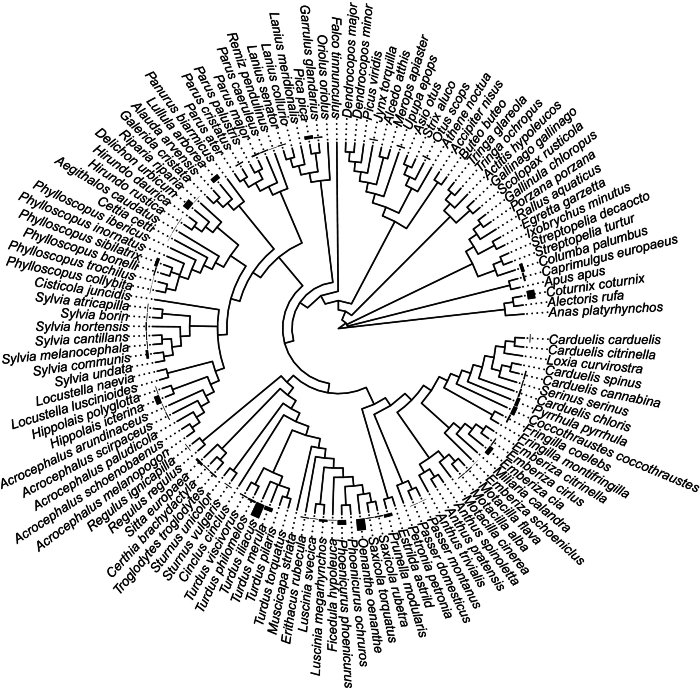
Phylogenetic tree of the species analysed together with a bar with the relative prevalence detected for each species. Each bar represents the relative tick prevalence per taxa (maximum = 0,03% for *Turdus iliacus,* see Supplementary Table for all the prevalences).

None of the life-history traits considered was significantly associated to tick prevalence when the phylogeny was included as a random factor (Fig. 1). Nevertheless, tick prevalence was significantly related to host phylogeny (post-mean = 5.19, CI-95% = 2.734–8.818), sampling location (post-mean = 1.07, CI-95% = 0.659–1.650) and hydrological year (post-mean = 0.04, CI-95% = 0.004–0.124). There was a clear and significant phylogenetic signal in the likelihood of being parasitized (λ = 0.699, CI-95% = 0.571–0.824, Fig. 2).

**Fig. 3:**
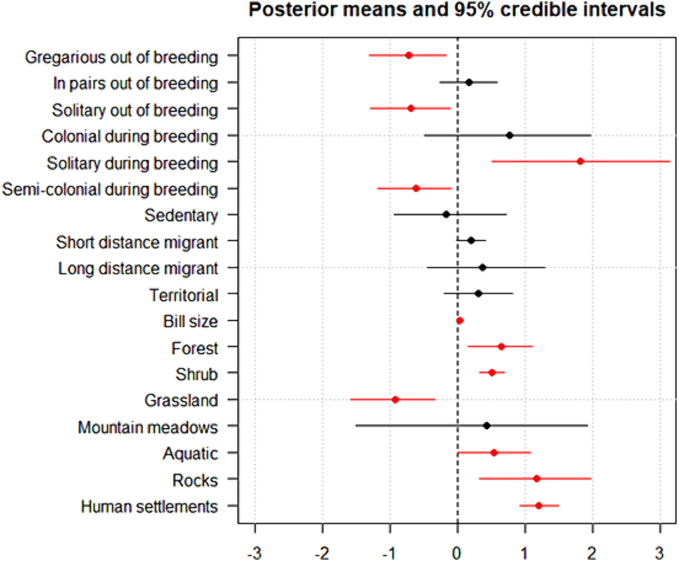
Posterior mean estimates and 95% credible intervals of fixed effects predictors of the probability of being infested by ticks when phylogeny is not included as a random factor. Parameters with intervals that do not overlap zero are considered to have a significant influence on the response and are plotted in red. Intercept were removed for visualisation. (For interpretation of the references to colour in this figure legend, the reader is referred to the web version of this article.)

When phylogeny was not included in the model, a number of life story traits appeared correlated to tick prevalence (Fig. 3). In particular, individuals from species which are gregarious or solitaries out of the breeding season had lower probabilities of having ticks. In the same way solitary breeding species had higher probabilities of having ticks while semi-colonial species have lower probabilities. Species living in forest, shrubs, rocks, human settlements or aquatic habitats had higher prevalences while species living in grasslands had lower prevalences. Finally, migratory behaviour was unrelated to tick prevalence while there was a positive relationship between bill size and tick prevalence. Both sampling location (post-mean = 1.10, CI-95% = 0.661–1.683) and hydrological year (post-mean = 0.04, CI-95% = 0.003–0.115) had a significant relationship with the probability of being infected by ticks.

## 4 Discussion

Tick population dynamics and dispersal through their host movements may have important impacts on the transmission dynamics of the pathogens they transmit [31]. Ticks present an aggregated distribution with a few individuals harbouring most of the ticks [12,32]. Those individuals heavily infected are considered superspreaders being responsible of ectoparasite population maintenance and playing an important role in pathogen transmission [33]. Consequently, identify vertebrate species or life-history traits associated to higher exposure to ticks is crucial to control and surveillance of tick populations and pathogens for early detection of outbreaks (e.g. virus Crimea-Congo in Spain [34]). When analysing tick prevalence we found important differences between localities and years, which reflect the large impact that climatic conditions had on tick distribution. In example, temperature determine latitudinal and altitudinal ranges for tick distribution while precipitation affects ticks abundance and reproduction cycle [8,10,35]. However, we must consider that the effect of localities is likely strongly correlated with the ringer identity, so further analysis must be done to properly understand the effects of the locality. Furthermore, the host phylogeny significantly influenced infection probability, with species within certain phylogenetic groups exhibiting higher probabilities of infestation. Consequently, ticks were more frequently observed in thrushes, quails, and finches. These groups with higher tick prevalence were mainly characterised by breeding solitarily, being gregarious out of the breeding season and habiting mainly forest, shrub and human settlements. These species also have smaller bills than the mean of all the species captured.

The overall prevalence found (0.14%, CI95%: 0.13–0.15) is lower than the previously reported in the few extensive avian studies done in Europe (e.g. 2.0% of 22,998 individuals, CI95%: 1.84–2.21 [36]; 7.3% of 9768 individuals, CI95%: 6.79–7.83 [8]; 3.01% of 23,949 individuals, CI95%: 2.81–3.25 [37]). These differences could be due to the lack of expertise of the citizen searching for ticks in our study, which can be less sensitive than instructed researchers [38]. Nevertheless, it was shown that once citizen becomes familiar with ectoparasites, the efficacy of sampling increases and may even be comparable to those of professional entomologist researchers [39]. Tick screening should comprise a rapid visual assessment for the presence of any ticks on bare body parts, especially around the eyes and beak of each bird [40]. Some life stages of ticks may be harder to detect, namely larvae and nymphae, while blood engorged adult females are more easily spotted. Although the estimated prevalence in our study is lower than in previous studies the ranking of species according to prevalence is similar to those reported in previous studies. The orders Passeriformes and Galliformes are among the ones with highest prevalence of ticks and also the species *T. merula*, *T. iliacus or P. phoenicurus* [8,37,41]. Thus, despite variations in absolute numbers, citizen science with an appropriate training could still provide valuable insights into ectoparasite studies.

After controlling for phylogeny, none of the life-history traits explained variation in tick prevalence. This lack of significance may be attributed to strong association that could exits between life-history traits and phylogeny. However, when excluding phylogeny several life-history traits arise as significant predictors of tick presence. One of such traits is host gregariousness, which is often linked to higher parasite prevalence as proximity and contact between individuals facilitate transmission, especially in contact-transmitted and low mobile parasites [42]. Although many ticks may actively search for hosts, mobility of some species and life stages are more reduced and may benefit from a shorter interindividual distance for dispersal. On the other hand, Brown & Brown [43] propose that in large colonies few individuals could harbour most of the parasites due to the differences in individual quality, with gregariousness leading to less parasitization. We found that the relationship between bird association and the probability of being infected by ticks varies depending not only on gregariousness but also on the period when the host association occurs, namely during the breeding season or outside of it.

During the breeding season solitary bird species are more frequently parasitized by ticks while semi-colonial are less frequently parasitized. Breeding season coincide with the period of higher abundance and mobility of ticks facilitating the contact between bird and ticks. Furthermore, in the breeding season, birds increase their activity in relation to pairing, nest-defence, foraging and nest location, being more frequent the contact between individuals and the exposition to different habitats. In this framework, bird species are prone to contact with both off-host and on-host ticks, but while solitary species cannot easily donate them, semi-colonial species could benefit of close contact with conspecific to share it and benefit if ticks aggregates on weaker individuals [43]. The absence of differences on gregarious species suggest a possible threshold in these relationships.

Regarding to their relationship out of the breeding season, species which are gregarious or solitaries are less frequently parasitized by ticks. Out of the breeding season, when conditions are harsher, ticks are less active and bird activities related with breeding disappear [44]. In this context, certain bird species are less likely to contact with off-ticks, so while gregarious species still benefit from dilution effect, solitary species benefit from scarce contact with on-host ticks.

Migratory behaviour is frequently associated with high parasite exposition, arguing that migratory species face larger diversity of environments [45]. But as other authors [31,37], we did not find support for the effect of the species migratory behaviour on the probability of being infested by ticks. Migration could act culling infected individuals and preventing parasite accumulation, preventing the apparition of such relationship [46]. However, the impact of migration on parasitism may vary among parasites according to the impact that those parasites has on their hosts and their migratory costs. The relationship between migratory behaviour and prevalence or intensity of parasitism may be largely determined by the impact that parasites have on individuals migratory capacity, and on the costs of migration [47]. When the costs of parasitism are high in relation to those of migration, a lower prevalence or intensity is expected in migrant species, when the costs of parasitism are low in relation to the costs of migration, a lower prevalence or intensity of infection may be expected in resident species [47].

Efficiency of bird grooming and preening are determinant in ectoparasite abundance [48]. We found that species with longer beaks have higher probabilities of being infested by ticks. Long beaks have been associated with lower preening efficiency but comparisons of host taxa with different beak sizes did not found differences in lice load [49]. We found support to longer bills as worse for preening as we found more prevalence of ticks in bird species with longer bills. Attached ticks could be more resistant to preening than other free-moving ectoparasites being more affected by the inefficiency of longer beaks.

Habitat used by different bird species is key in the differences in tick prevalence. Ticks are non-permanent ectoparasite which spent more than 90% of their life off-host being strongly stressed by environmental factors [50]. Moisture and temperature are key environmental variables determining tick survival and activity [51], as desiccation and water balance maintenance is critical for them [52]. Shelter-seeking behaviour to prevent dissection is a common strategy of ticks which help them to cope with harsh conditions. We found that bird species using grassland were less likely of being infected by ticks. Grasslands are open habitats where the plant cover is dominated by grasses. In this kind of habitats higher sun exposition or lack of shelter could lead to lower tick populations. Some studies have also found lower abundance of ticks in grasslands than in forests [53,54]. The probability of being infested by tick was higher in bird species associated with shrubs, rocky and aquatic habitats and human settlements which could be associated to higher shelter possibilities and higher moisture. It is particularly relevant that birds who use human settlements are one of the most frequently infested with ticks. It is important to underline the implications that this may have for the risk of spillover of ticks to humans and domestic animals and the maintenance of tick and pathogen populations close to humans.

## 5 Conclusions

Citizen science has emerged as a valuable method for investigating the occurrence of ectoparasites in wildlife. Nevertheless, they also have some limitations and data should be explored cautiously, citizens' observations have been shown to be less accurate than scientists' ones [38] or more variable [55]. Data quality is a significant concern in many citizen science programs [56], although some studies have shown promising results [57].

In this study, we found some data quality challenges, but the participation of the ringer collective with high training capacity provides greats opportunities for rapid improvement. How to improve the program? Our results suggest that probably the tick detection is highly biased towards engorged adults that are more easily detected. To address this issue, it is necessary to provide training to the ringers to enhance their ability to detect immature stages and provide them skills to collect ticks from birds for taxonomic identification. This is an important limitation of our current study, because no taxonomic information is available for the detected ticks. Providing training to the ringers for the detection but also the safely removal and preservation of the ticks may provide two important complementary benefits: first by allowing the identification of the life stages and taxonomy of the detected ticks, and second by allowing direct pathogen screening in the collected ticks further improving the usefulness of the ringing activity. Cooperation between ringing centrals, research institutes and animal and public health authorities may generate synergies providing a source of knowledge and biological material for surveillance purposes.

While countries like United States and Italy have already implemented citizen science programs involving general public in the collection of ticks but to the best of our knowledge none has involved ringers yet [58,59]. By involving ringers in tick surveillance, we can expand the scope of the program and benefit from their expertise. Species such as thrushes, finches and quails were among the species with a higher prevalence of ticks and are excellent candidates for tick surveillance of ticks and monitoring the potential pathogens they may transmit.

## Funding

This study was funded by MCIN through the European Regional Development Fund Sustainability for Mediterranean Hotspots in Andalusia (SUMHAL), [LIFEWATCH-2019-09-CSIC-4, POPE 2014–2020]. JF was supported by the project PLEC2021–007968 (NEXTHREAT) funded by MCIN (AEI/10.13039/5011000110333). JV was supported by a contract associated to the project SUMHAL during the preparation of this study and receive financial support from the Margarita Salas program (funded by Spanish Ministry of Universities, the European Union-NextGenerationEU, and the University of Granada) and Juan de la Cierva program (Ref. FJC2021–048057-I, funded by MICIU/AEI/10.13039/501100011033 and the European Union NextGenerationEU/PRTR). The ICO Bird Ringing Office counts with the financial support of the Catalan Government.

## CRediT authorship contribution statement

**Jesus Veiga:** Data curation, Formal analysis, Investigation, Methodology, Validation, Writing – review & editing. **Oriol Baltà:** Data curation, Investigation, Supervision, Validation, Writing – review & editing. **Jordi Figuerola:** Conceptualization, Funding acquisition, Investigation, Methodology, Project administration, Resources, Supervision, Validation, Writing – review & editing.

## Declaration of competing interest

The authors of this manuscript declare that there are no conflict of interests regarding the publication of this article.

Jesús Veiga, first corresponding author of the article, signing on behalf of all coauthors of the article.

## Data Availability

The data of tick presence on each individual bird used in the analysis are available through GBIF.

## Appendix A Supplementary data

Number of individuals captured of each species, together with tick infestation parameters and their life-history traits Image 1
