# Supplementary material for: Does bird life-history influence the prevalence of ticks? A citizen science study in North East Spain
Source: One Health. 2024 Apr 3;18:100718. doi: 10.1016/j.onehlt.2024.100718 (PMC11026695; doi:10.1016/j.onehlt.2024.100718)
Supplement: Supplementary file 1 — Number of individuals captured of each species, together with tick infestation parameters and their life-history traits [file mmc1.docx]

**Supplementary material**

| **Species** | **Order** | **Total of individuals** | **Number of infested** | **Prevalence** | **Confidence interval 95%** | **Bill size (mm)** | **Association during nesting** | **Association outside the breeding season** | **Territoriality** | **Migratory behaviour** | **Habitat** |
| --- | --- | --- | --- | --- | --- | --- | --- | --- | --- | --- | --- |
| *Accipiter nisus* | Accipitriformes | 69 | 0 | 0 | NA | 12.900 | S | SO | Yes | SD | F |
| *Buteo buteo* | Accipitriformes | 39 | 0 | 0 | NA | 22.300 | S | SO | Yes | SE; SD | F |
| *Anas platyrhynchos* | Anseriformes | 103 | 0 | 0 | NA | 53.600 | S | GR | No | SD | A |
| *Apus apus* | Caprimulgiformes | 35 | 0 | 0 | NA | 6.600 | C | GR | Yes | LD | R; H |
| *Caprimulgus europaeus* | Caprimulgiformes | 295 | 1 | 0.0034 | 0.1137 | 8.850 | S | SO | Yes | LD | F |
| *Actitis hypoleucos* | Charadriiformes | 100 | 0 | 0 | NA | 24.550 | S | SO | Yes | LD | A |
| *Gallinago gallinago* | Charadriiformes | 82 | 0 | 0 | NA | 67.550 | S | GR | Yes | SD; LD | A |
| *Scolopax rusticola* | Charadriiformes | 16 | 0 | 0 | NA | 72.600 | S | SO | No | SD | F; A |
| *Tringa glareola* | Charadriiformes | 18 | 0 | 0 | NA | 28.800 | S | GR | No | LD | F; A |
| *Tringa ochropus* | Charadriiformes | 29 | 0 | 0 | NA | 34.550 | S | SO | Yes | SD; LD | F; A |
| *Columba palumbus* | Columbiformes | 147 | 1 | 0.0068 | 0.1606 | 21.250 | S | GR | Yes | SD | F; H |
| *Streptopelia decaocto* | Columbiformes | 257 | 0 | 0 | NA | 16.750 | S | GR | Yes | SE | H |
| *Streptopelia turtur* | Columbiformes | 80 | 0 | 0 | NA | 16.400 | S | PA; SO | No | LD | F |
| *Alcedo atthis* | Coraciiformes | 4535 | 0 | 0 | NA | 43.750 | S | SO | Yes | SE; SD | A |
| *Merops apiaster* | Coraciiformes | 407 | 0 | 0 | NA | 42.150 | C | GR | No | LD | F; S; G |
| *Upupa epops* | Coraciiformes | 443 | 1 | 0.0023 | 0.0929 | 57.850 | S | SO | Yes | LD | F; G |
| *Falco tinnunculus* | Falconiformes | 69 | 0 | 0 | NA | 14.500 | S | SO | No | LD | G; H |
| *Alectoris rufa* | Galiiformes | 24 | 0 | 0 | NA | 13.600 | S | GR | Yes | SE | G; M; R |
| *Coturnix coturnix* | Galiiformes | 61 | 1 | 0.0164 | 0.2468 | 8.950 | S | SO; GR | Yes | LD | G |
| *Gallinula chloropus* | Gruiformes | 214 | 0 | 0 | NA | 25.400 | S | SO | Yes | SE; SD | F; A |
| *Porzana porzana* | Gruiformes | 31 | 0 | 0 | NA | 19.050 | S | SO | Yes | LD | A |
| *Rallus aquaticus* | Gruiformes | 177 | 0 | 0 | NA | 39.200 | S | SO | Yes | SE; SD | A |
| *Acrocephalus arundinaceus* | Passeriformes | 5266 | 3 | 0.0006 | 0.0156 | 23.150 | S | SO | Yes | LD | A |
| *Acrocephalus melanopogon* | Passeriformes | 988 | 0 | 0 | NA | 15.150 | S | SO | Yes | SE; SD | A |
| *Acrocephalus paludicola* | Passeriformes | 32 | 0 | 0 | NA | 13.650 | S | SO | No | LD | A |
| *Acrocephalus schoenobaenus* | Passeriformes | 2694 | 0 | 0 | NA | 14.700 | S | SO | Yes | LD | S; A |
| *Acrocephalus scirpaceus* | Passeriformes | 68,685 | 86 | 0.0013 | 0.0008 | 17.050 | S | SO | Yes | LD | A |
| *Aegithalos caudatus* | Passeriformes | 6216 | 2 | 0.0003 | 0.0176 | 7.475 | SC | GR | No | SE | F; S |
| *Alauda arvensis* | Passeriformes | 82 | 0 | 0 | NA | 15.200 | S | GR; SO | Yes | SE; SD | G; M |
| *Anthus pratensis* | Passeriformes | 513 | 0 | 0 | NA | 14.450 | S | GR | Yes | SD | G; M |
| *Anthus spinoletta* | Passeriformes | 1321 | 0 | 0 | NA | 16.650 | S | SO; GR | Yes | SD | M |
| *Anthus trivialis* | Passeriformes | 180 | 0 | 0 | NA | 15.100 | S | SO | Yes | LD | F |
| *Carduelis cannabina* | Passeriformes | 406 | 1 | 0.0025 | 0.0970 | 12.800 | S; SC | GR | Yes | SE; SD | F; S; M |
| *Carduelis carduelis* | Passeriformes | 4743 | 4 | 0.0008 | 0.0142 | 15.500 | S; SC | GR | Yes | SE | F; H |
| *Carduelis chloris* | Passeriformes | 6895 | 21 | 0.0030 | 0.0051 | 16.650 | S | GR | No | SE | F; S; H |
| *Carduelis citrinella* | Passeriformes | 103 | 0 | 0 | NA | 11.700 | SC | GR | Yes | SD | F; S; M |
| *Carduelis spinus* | Passeriformes | 1371 | 0 | 0 | NA | 12.950 | S | GR | Yes | SD | F |
| *Certhia brachydactyla* | Passeriformes | 2367 | 3 | 0.0013 | 0.0232 | 17.650 | S | SO | Yes | SE | F |
| *Cettia cetti* | Passeriformes | 20,912 | 2 | 0.0001 | 0.0096 | 14.050 | S | SO | Yes | SE | S; A |
| *Cinclus cinclus* | Passeriformes | 19 | 0 | 0 | NA | 21.750 | S | SO | Yes | SE | A |
| *Cisticola juncidis* | Passeriformes | 1040 | 0 | 0 | NA | 12.100 | S | SO | Yes | SE | G; A |
| *Coccothraustes coccothraustes* | Passeriformes | 445 | 4 | 0.0090 | 0.0460 | 24.850 | S; C | GR | Yes | SE; SD | F |
| *Delichon urbicum* | Passeriformes | 302 | 4 | 0.0132 | 0.0556 | 9.850 | C | GR | Yes | LD | R; H |
| *Emberiza cia* | Passeriformes | 423 | 4 | 0.0095 | 0.0472 | 13.500 | S | SO; GR | Yes | SE | S; R |
| *Emberiza cirlus* | Passeriformes | 1588 | 3 | 0.0019 | 0.0283 | 14.150 | S | GR | Yes | SE | F; S |
| *Emberiza citrinella* | Passeriformes | 49 | 0 | 0 | NA | 14.000 | S | GR | Yes | SE; SD | F; S; G |
| *Emberiza schoeniclus* | Passeriformes | 26,843 | 3 | 0.0001 | 0.0069 | 12.000 | S | GR | Yes | SE; SD | A |
| *Erithacus rubecula* | Passeriformes | 32,613 | 49 | 0.0015 | 0.0015 | 14.500 | S | SO | Yes | SE; SD | F; S |
| *Estrilda astrild* | Passeriformes | 218 | 0 | 0 | NA | 9.850 | S | GR | Yes | SE | A |
| *Ficedula hypoleuca* | Passeriformes | 5610 | 3 | 0.0005 | 0.0151 | 11.800 | S | SO | Yes | LD | F |
| *Fringilla coelebs* | Passeriformes | 7083 | 21 | 0.0030 | 0.0051 | 15.000 | S | GR | Yes | SE; SD | F; H |
| *Fringilla montifringilla* | Passeriformes | 145 | 0 | 0 | NA | 15.500 | SC | GR | Yes | SD | F |
| *Galerida cristata* | Passeriformes | 92 | 0 | 0 | NA | 20.700 | S | PA | Yes | SE | G |
| *Garrulus glandarius* | Passeriformes | 878 | 2 | 0.0023 | 0.0467 | 33.400 | S | PA | Yes | SE | F |
| *Hippolais icterina* | Passeriformes | 91 | 1 | 0.0110 | 0.2032 | 16.550 | S | SO | Yes | LD | F; S |
| *Hippolais polyglotta* | Passeriformes | 5618 | 4 | 0.0007 | 0.0131 | 15.800 | S | SO | Yes | LD | F; S |
| *Hirundo daurica* | Passeriformes | 37 | 0 | 0 | NA | 11.050 | S; C | GR | No | LD | R |
| *Hirundo rustica* | Passeriformes | 20,391 | 7 | 0.0003 | 0.0052 | 12.250 | S | GR | Yes | LD | G; H |
| *Lanius collurio* | Passeriformes | 149 | 0 | 0 | NA | 18.350 | S | SO | Yes | LD | S |
| *Lanius meridionalis* | Passeriformes | 26 | 0 | 0 | NA | 23.150 | S | SO | Yes | SE; SD | F; S |
| *Lanius senator* | Passeriformes | 662 | 1 | 0.0015 | 0.0761 | 18.500 | S | SO | Yes | LD | F |
| *Locustella luscinioides* | Passeriformes | 1081 | 0 | 0 | NA | 15.700 | S | SO | Yes | LD | A |
| *Locustella naevia* | Passeriformes | 437 | 0 | 0 | NA | 14.300 | S | SO | Yes | LD | S; G; A |
| *Loxia curvirostra* | Passeriformes | 70 | 0 | 0 | NA | 22.600 | S | GR | Yes | SE | F |
| *Lullula arborea* | Passeriformes | 100 | 1 | 0.0100 | 0.1940 | 14.600 | S | GR | Yes | SE; SD | F; S; G |
| *Luscinia megarhynchos* | Passeriformes | 9091 | 40 | 0.0044 | 0.0032 | 17.100 | S | SO | Yes | LD | F; S |
| *Luscinia svecica* | Passeriformes | 4667 | 3 | 0.0006 | 0.0166 | 15.650 | S | SO | Yes | LD | F; S; A |
| *Miliaria calandra* | Passeriformes | 634 | 0 | 0 | NA | 16.050 | S | GR | Yes | SE | G |
| *Motacilla alba* | Passeriformes | 1060 | 1 | 0.0009 | 0.0601 | 15.950 | S | GR; SO | Yes | SE; SD | G; A; H |
| *Motacilla cinerea* | Passeriformes | 828 | 0 | 0 | NA | 16.000 | S | GR; SO | Yes | SD | S; A |
| *Motacilla flava* | Passeriformes | 1530 | 2 | 0.0013 | 0.0354 | 16.050 | S | GR | Yes | LD | S; G; A |
| *Muscicapa striata* | Passeriformes | 994 | 0 | 0 | NA | 15.450 | S | SO | Yes | LD | F; H |
| *Oenanthe oenanthe* | Passeriformes | 41 | 1 | 0.0244 | 0.2986 | 17.300 | S | SO | Yes | LD | G; M; R |
| *Oriolus oriolus* | Passeriformes | 493 | 0 | 0 | NA | 27.800 | S | SO | Yes | LD | F |
| *Panurus biarmicus* | Passeriformes | 22 | 0 | 0 | NA | 10.700 | SC | GR | No | SE | A |
| *Parus ater* | Passeriformes | 1179 | 0 | 0 | NA | 10.850 | S | GR | Yes | SE | F |
| *Parus caeruleus* | Passeriformes | 8154 | 16 | 0.0020 | 0.0054 | 9.050 | S | GR | Yes | SE | F; H |
| *Parus cristatus* | Passeriformes | 955 | 2 | 0.0021 | 0.0448 | 11.200 | S | GR | Yes | SE | F; |
| *Parus major* | Passeriformes | 9747 | 29 | 0.0030 | 0.0037 | 12.500 | S | GR | Yes | SE | F; H |
| *Parus palustris* | Passeriformes | 101 | 0 | 0 | NA | 10.200 | S | PA | Yes | SE | F |
| *Passer domesticus* | Passeriformes | 15,861 | 6 | 0.0004 | 0.0064 | 15.550 | C | GR | No | SE | F; S; H |
| *Passer montanus* | Passeriformes | 3894 | 1 | 0.0003 | 0.0314 | 13.200 | C | GR | No | SE | F; S |
| *Petronia petronia* | Passeriformes | 106 | 0 | 0 | NA | 17.150 | S; C | GR | Yes | SE | S; G; M; R |
| *Phoenicurus ochruros* | Passeriformes | 999 | 0 | 0 | NA | 15.050 | S | SO; PA | Yes | SE; SD | R; H |
| *Phoenicurus phoenicurus* | Passeriformes | 2369 | 24 | 0.0101 | 0.0081 | 14.600 | S | SO; PA | Yes | LD | F |
| *Phylloscopus bonelli* | Passeriformes | 1000 | 2 | 0.0020 | 0.0437 | 12.550 | S | SO | Yes | LD | F |
| *Phylloscopus collybita* | Passeriformes | 35,513 | 13 | 0.0004 | 0.0029 | 11.150 | S | SO | Yes | SD; LD | F |
| *Phylloscopus ibericus* | Passeriformes | 19 | 0 | 0 | NA | 11.150 | S | SO | Yes | SD; LD | F |
| *Phylloscopus inornatus* | Passeriformes | 15 | 0 | 0 | NA | 10.950 | S | SO; GR | Yes | LD | F |
| *Phylloscopus sibilatrix* | Passeriformes | 468 | 3 | 0.0064 | 0.0520 | 12.900 | S | SO | Yes | LD | F |
| *Phylloscopus trochilus* | Passeriformes | 13,886 | 2 | 0.0001 | 0.0118 | 11.800 | S | SO; GR | Yes | LD | F; S |
| *Pica pica* | Passeriformes | 143 | 1 | 0.0070 | 0.1628 | 39.100 | S | SO; GR | Yes | SE | F; H |
| *Prunella modularis* | Passeriformes | 3092 | 20 | 0.0065 | 0.0078 | 14.600 | S | SO | Yes | SE; SD | F; S |
| *Pyrrhula pyrrhula* | Passeriformes | 278 | 2 | 0.0072 | 0.0825 | 13.900 | S | SO; PA; GR | No | SE; SD | F |
| *Regulus ignicapilla* | Passeriformes | 2766 | 2 | 0.0007 | 0.0263 | 11.000 | S | GR | Yes | SE; SD | F |
| *Regulus regulus* | Passeriformes | 226 | 0 | 0 | NA | 10.450 | S | GR | Yes | SE; SD | F |
| *Remiz pendulinus* | Passeriformes | 6946 | 3 | 0.0004 | 0.0136 | 11.400 | S | GR | Yes | SD | A |
| *Riparia riparia* | Passeriformes | 5099 | 1 | 0.0002 | 0.0274 | 9.900 | C | GR | No | LD | A |
| *Saxicola rubetra* | Passeriformes | 508 | 1 | 0.0020 | 0.0868 | 14.800 | S | SO | Yes | LD | G |
| *Saxicola torquatus* | Passeriformes | 1580 | 2 | 0.0013 | 0.0348 | 15.250 | S | SO; PA | Yes | SE; SD | S |
| *Serinus serinus* | Passeriformes | 5546 | 9 | 0.0016 | 0.0088 | 10.500 | S; SC | GR | Yes | SE; SD | F; H |
| *Sitta europaea* | Passeriformes | 212 | 1 | 0.0047 | 0.1340 | 20.200 | S | PA | Yes | SE | F |
| *Sturnus unicolor* | Passeriformes | 598 | 0 | 0 | NA | 29.200 | S; SC | GR | Yes | SE | F; G; H |
| *Sturnus vulgaris* | Passeriformes | 2599 | 3 | 0.0012 | 0.0222 | 28.850 | C | GR | Yes | SE; SD | F; G; H |
| *Sylvia atricapilla* | Passeriformes | 46,285 | 32 | 0.0007 | 0.0016 | 14.450 | S | SO | Yes | SE; SD | F |
| *Sylvia borin* | Passeriformes | 4433 | 3 | 0.0007 | 0.0170 | 14.500 | S | SO | Yes | LD | F; S |
| *Sylvia cantillans* | Passeriformes | 3447 | 5 | 0.0015 | 0.0149 | 12.850 | S | PA; GR | Yes | LD | F; S |
| *Sylvia communis* | Passeriformes | 1948 | 10 | 0.0051 | 0.0140 | 14.000 | S | SO | Yes | LD | F; S |
| *Sylvia hortensis* | Passeriformes | 872 | 2 | 0.0023 | 0.0468 | 17.800 | S | GR | Yes | LD | F; S |
| *Sylvia melanocephala* | Passeriformes | 10,565 | 11 | 0.0010 | 0.0057 | 14.200 | S | SO; PA | Yes | SE | S |
| *Sylvia undata* | Passeriformes | 312 | 0 | 0 | NA | 12.750 | S | SO; PA | Yes | SE | F; S |
| *Troglodytes troglodytes* | Passeriformes | 1950 | 5 | 0.0026 | 0.0198 | 13.650 | S | SO | Yes | SE; SD | F |
| *Turdus iliacus* | Passeriformes | 98 | 3 | 0.0306 | 0.1108 | 14.450 | S | GR | Yes | SD | F; S |
| *Turdus merula* | Passeriformes | 15,573 | 127 | 0.0082 | 0.0014 | 17.200 | S | GR | Yes | SE; SD | F; S; H |
| *Turdus philomelos* | Passeriformes | 5782 | 22 | 0.0038 | 0.0055 | 14.250 | S | SO | Yes | SE; SD | F; S; H |
| *Turdus pilaris* | Passeriformes | 19 | 0 | 0 | NA | 15.600 | C; S | GR | Yes | SD | F |
| *Turdus torquatus* | Passeriformes | 23 | 0 | 0 | NA | 16.500 | S | GR | Yes | SD | F; G; R |
| *Turdus viscivorus* | Passeriformes | 484 | 1 | 0.0021 | 0.0889 | 15.700 | S | GR | Yes | SE; SD | F |
| *Egretta garzetta* | Pelecaniformes | 18 | 0 | 0 | NA | 87.950 | C | GR; SO | Yes | LD | G; A |
| *Ixobrychus minutus* | Pelecaniformes | 388 | 0 | 0 | NA | 47.550 | S | SO; PA | Yes | LD | A |
| *Dendrocopos major* | Piciformes | 395 | 0 | 0 | NA | 28.150 | S | SO | Yes | SE | F; H |
| *Dendrocopos minor* | Piciformes | 93 | 0 | 0 | NA | 17.300 | S | SO | Yes | SE | F |
| *Jynx torquilla* | Piciformes | 594 | 1 | 0.0017 | 0.0803 | 15.950 | S | SO | Yes | LD | F |
| *Picus viridis* | Piciformes | 273 | 0 | 0 | NA | 43.700 | S | SO | Yes | SE | F; |
| *Asio otus* | Strigiformes | 22 | 0 | 0 | NA | 28.150 | S | SO | Yes | SE | F |
| *Athene noctua* | Strigiformes | 50 | 0 | 0 | NA | 20.500 | S | SO; PA | Yes | SE | F; H |
| *Otus scops* | Strigiformes | 440 | 1 | 0.0023 | 0.0932 | 18.200 | S | SO | Yes | LD | F |
| *Strix aluco* | Strigiformes | 28 | 0 | 0 | NA | 31.250 | S | SO; PA | Yes | SE | F; H |

Supplementary table 1. Number of individuals captured of each species and order (sorted alphabetically by order) together with the number of individuals infested by ticks, the tick prevalence for each species and the 95% confidence interval. Life-history traits of mean bill size (in mm), migration behaviour (SE=sedentary; SD=short-distance migrant; LD= long-distance migrant), gregariousness out of the breeding season (GR= gregarious; PA= in pairs; SO=solitary) and in the nesting period (C= colonial; SC= semicolonial; S= solitary), territoriality and main habitat occupied in the breeding area (F= forest; S= shrub; G= grassland; M= mountain meadows; A=aquatic; R= rocks; H= human settlements) was also showed.
